# Prevention of Deficit in Neuropsychiatric Disorders through Monitoring of Arsenic and Its Derivatives as Well as Through Bioinformatics and Cheminformatics

**DOI:** 10.3390/ijms20081804

**Published:** 2019-04-12

**Authors:** Speranta Avram, Ana Maria Udrea, Adina Negrea, Mihaela Ciopec, Narcis Duteanu, Carmen Postolache, Corina Duda-Seiman, Daniel Duda-Seiman, Sergey Shaposhnikov

**Affiliations:** 1 Faculty of Biology, University of Bucharest, Splaiul Independentei 91-95, 050095 Bucharest, Romania; carmen_postolache83@yahoo.com; 2 National Institute for Laser Plasma and Radiation Physics, Atomistilor Street 409, 077125 Magurele, Romania; m.a.u.anamaria@gmail.com; 3 Politehnica University of Timisoara, Faculty of Industrial Chemistry and Environmental Engineering, Piata Victoriei, 2, 300006 Timisoara, Romania; 4 Faculty of Chemistry, Biology, Geography, West University of Timișoara, I.H.Pestalozzi 16, 300115 Timisoara, Romania; cori_mam@yahoo.com; 5 University of Medicine and Pharmacy "Victor Babes, Timişoara, Eftimie Murgu Square 2, 300041 Timisoara, Romania; 6 Norgenotech AS, Gaustadalleen 21, 0349 Oslo, Norway; sas@norgenotech.no

**Keywords:** arsenic, brain, bioinformatics, cheminformatics, nanoparticles

## Abstract

Neuropsychiatric disorders are induced by various risk factors, including direct exposure to environmental chemicals. Arsenic exposure induces neurodegeneration and severe psychiatric disorders, but the molecular mechanisms by which brain damage is induced are not yet elucidated. Our aim is to better understand the molecular mechanisms of arsenic toxicity in the brain and to elucidate possible ways to prevent arsenic neurotoxicity, by reviewing significant experimental, bioinformatics, and cheminformatics studies. Brain damage induced by arsenic exposure is discussed taking in account: the correlation between neuropsychiatric disorders and the presence of arsenic and its derivatives in the brain; possible molecular mechanisms by which arsenic induces disturbances of cognitive and behavioral human functions; and arsenic influence during psychiatric treatments. Additionally, we present bioinformatics and cheminformatics tools used for studying brain toxicity of arsenic and its derivatives, new nanoparticles used as arsenic delivery systems into the human body, and experimental ways to prevent arsenic contamination by its removal from water. The main aim of the present paper is to correlate bioinformatics, cheminformatics, and experimental information on the molecular mechanism of cerebral damage induced by exposure to arsenic, and to elucidate more efficient methods used to reduce its toxicity in real groundwater.

## 1. Arsenic Exposure Induces the Neuropsychiatric Disorders—General Aspects

Increased exposure of humans to higher levels of metals represents a serious concern for public health and, from these metals, lead (Pb), arsenic (As), and manganese (Mn) are considered biomarkers and are studied and proposed for detection because of their severe adverse health effects. It is important to note that, generally, from the literature to date, people are exposed to one, as opposed to all of the metals: this limits the real-life exposure scenarios [1].

Currently, arsenic is considered the most ubiquitous environmental pollutant and due to this, subsequently, the U.S. Environmental Protection Agency and Agency for Toxic Substances and Disease Registry [2] mention arsenic on the U.S. Priority List of Hazardous Substances [3].

The presence of arsenic in the environment has become a major problem for the world, especially in Bangladesh, India, China, and Japan [4,5]. Bangladesh and some Indian provinces, such as Bengal, are the most affected regions of the world in which more than 100 million people drink water contaminated with arsenic, and 21% of reported deaths are associated with arsenic poisoning [3].

Many types of minerals existing naturally in the Earth’s crust are responsible for arsenic release in water [6]. While the arsenic concentration in natural water is very low, in some areas it is sufficiently high (between 100–500 mg L^−1^) to be necessary to trigger alarms [7,8]. Many industries release arsenic-contaminated waters without adequate treatment, and in various regions the antropic source represents a major source for a higher concentration of arsenic and its derivatives in groundwater [4].

For this reason, the removal of arsenic from water is an important and urgent preoccupation around the world and, at present, many techniques have been developed for removing arsenic (e.g., precipitation, filtration, solvent extraction, bioremediation, and widely used-absorption) [9]. Recently, many types of absorbents have been developed [9,10,11]. Techniques based on reverse osmosis and electrodialysis [10] are generally expensive and have a low efficiency if not performed in advance to oxidize As(III) to As(V), but are more efficient in removing As(V) [11].

A systematic review regarding the pollutant effects of chemicals, such as bisphenol A, polycyclic aromatic hydrocarbons (PAHs), polyfluoroalkyl chemicals, and metals on the nervous system was published by Avram et al. [12]. It was mentioned that the presence of pollutants is associated with the presence of several severe psychiatric disorders, such as ADHD [13,14], autism [15], schizophrenia [16], anxiety [17], and depression [18]. It was mentioned that human arsenic exposure may induce genotoxicity and cancer and is harmful for the liver, skin, kidneys, and the central nervous system. Arsenic exposure is known as a risk factor for developing Alzheimer’s and Parkinson’s diseases [19], but also the presence of arsenic could affect the whole body.

Recent publications discussed the chemical complexity of arsenic due to its multitude of compounds and reported arsenic toxicities and oxidation states in inducing neurodegeneration or others psychiatric disorders. Sub-chronic arsenic exposure is linked to neurodegeneration, which represents the main cause of cognitive disturbances, relevant for many neuropsychiatric disorders. Unfortunately, there are limited data regarding the effects of sub-chronic exposure to arsenic-contaminated drinking water on neuropsychiatric disorders and its mechanisms are able to induce a low activity in the prefrontal cortex [20].

Chia-Yu Chang et al. [21] mentioned that sub-chronic exposure to arsenic from drinking water (10 mg L^−1^ As_2_O_3_) represents a key factor in induced anxiety-like symptoms on normal mice for four weeks and intensified the depression symptoms in depressive mice after eight weeks. The symptomatology of depressive behavior was evaluated with a tail suspension test (TST) and forced swimming test (FST) on normal and depressive chemically-induced models, with and without arsenic-contaminated water [21]. The study results showed that, during eight weeks, the mice with chemically-induced depression, by drinking arsenic-contaminated water, presented statistically significant worse performances in both tests compared to other test groups [20].

Another important result from this study suggested that arsenic exposure has a high impact in neurodegeneration in the prefrontal cortex by reducing the number of proliferating cell nuclear antigens (PCNAs). It was mentioned that the number of PCNA-positive cells was (i) in normal mice, 32.5 ± 2.9; (ii) in normal mice with arsenic exposure, 16.5 ± 1.4; (iii) in the chemically-induced depression lot, 12 ± 2.6; and (iv) in the lot of mice with chemically-induced depression and arsenic exposure, 3.6 ± 2.05. Behavioral patterns of depression and anxiety involved the inhibition of serotonin receptor 5-HT subtype 1A. Chang et al. [21] showed that after eight weeks of arsenic exposure only the group of mice with chemically-induced depression and exposed to arsenic showed the decreased levels of the 5-HT subtype 1A receptor [20].

Denise Hill’s study [22] was concerned about the effect of common environmental toxicants including mercury (Hg), lead (Pb), manganese (Mn), arsenic (As), and cadmium (Cd) on autism spectrum disorders. Recent information has mentioned that prenatal manganese and lead exposure represents a serious cause of adverse neurobehavioral development in progeny. In their work, the authors analyzed in utero exposure to these toxicants, during the neuronal developmental stage. It was mentioned that the exposure to the brain of these metals leads to behavioral abnormalities and these changes are persistent in adult life [22].

Dickerson et al. [23] indicated that in districts with high concentrations of mercury and arsenic in the air, the rate of autism spectrum disorders are increased. The study was performed using data from five locations of Autism and Developmental Disabilities Monitoring (ADDM) during 2000–2008. The results showed that the incidence of pathologies from autism spectrum disorders is linked with the proximity of industrial areas with reported emissions of arsenic, lead, and mercury in the air [23].

Another study published in 2016 by Rodrigues et al. [24] mentioned that: (i) drinking arsenic-contaminated water is connected with decreased cognitive scores for children from Pabna (Bangladesh); and (ii) the high concentration of lead in blood was also associated with a decrease of cognitive scores using the Bayley Scales of Infant and Toddler Development, Third Edition (BSID-III) [25]. This study was performed on 524 children exposed to lead- and arsenic-contaminated water in pre- and perinatal life. The study results do not confirm an association between arsenic levels in blood and motor and language scores [24]. We selected from this work a suggestive table (Table 1) in which are presented the results revealed by the multivariate model between As, Mn, and Pb exposures and BSID-III scores at 20–40 months.

Katherine von Stackelberg et al. [26] reviewed the effects of prenatal and perinatal exposure to lead, arsenic, cadmium, and manganese and their subsequent effects on neurodevelopment. In this review it was mentioned that the high concentration of arsenic and lead in blood or urine was linked with autism behavior and cognition changes [26]. Stackelber’s work [27] mentioned that the long-time exposure at arsenic induced in the human body: ”(i) autism by down-regulating the CAT gene (catalase gene) which is involved in immune system response to oxidative stress; (ii) intellectual disabilities and autism by down-regulating of BDNF (*brain-derived neurotrophic factor*); (iii) various learning disorders which are linked by up--regulation of MAPK1 and MAPK3 (mitogen activated protein-kinases); (iv) up--regulates IL1B (mediator of inflammatory response)—linked to learning disorders; (v) increases the level of serotonin and decreasing norephinephrine associated with apoptosis or apoptotic markers in astrocytes”. It was also mentioned by Chou et al. [28] that arsenite down-regulates the expression of brain-derived neurotrophic factor, one of the critical factors involved in memory and learning from neuroblastoma SH-SY5Y cells.

## 2. Possible Molecular Mechanisms of Arsenic-Induced Disturbance of Cognitive Human Functions

There are mentioned few possible mechanisms by which arsenic and other metals are able to induce neuropsychiatric disorders. It was mentioned that arsenic has the ability to easily cross the blood brain barrier, affecting basal ganglia, the hippocampus, and cortex [1].

It was reported that few chemicals could inhibit the acetylcholine esterase causing neurotoxicity by accumulation of acetylcholine in the synaptic cleft, thus being at the root of overstimulation of nicotinic and muscarinic receptors. Lipid peroxidation caused by oxidative stress may alter the enzyme’s activity [29,30,31]. More so, acetylcholine and dopamine functions are affected by the presence of arsenic [32,33,34].

There are several studies [24,35,36,37] which reported that arsenic exposure could disturb attention and memory processes. The inorganic form of arsenic, known as sodium arsenite, is linked to impairing memory and learning in pregnant dams and future offspring [38]. The effects of sodium arsenite were studied on spatial memory and it was shown that a daily dose of 15 mg kg^−1^ greatly affected the performance in the Y maze test and Morris water maze task [39]. The impaired learning and memory performance noticed in rats exposed to arsenic may be explained by decreased expression of mRNA glutamate receptor [40]. It was reported that arsenic metabolites permeated the blood brain barrier and are accumulated in the hippocampus creating cytotoxicity. Additionally, the arsenic exposure decreased expression of 2A subunit belonging to *N*-methyl-d-aspartate receptor gene, from the rat hippocampus alongside with phosphorylated-Ca^2+^/calmodulin-independent protein kinase II alpha and postsynaptic density protein 95 [41]. More so, the protein expression of synaptic GT*P*-ase which is a negative regulator of mitogen-activated protein kinases had an increase in activity [42]. 

Few studies presented the ability of arsenic to modify the structure of hippocampal neurons by inducing the pathological alterations of endothelial cells and neurons [41,43].

Recently, Bao-Fei Sun et al. [44] published a paper regarding the arsenic effect in memory, by tested the negative effects of arsenic exposure of mice in their long-term memory and in their ability for object recognition. Different doses of arsenic (e.g., 0, 1, 3, and 10 mg kg^−1^) were administered on a daily basis for 12 weeks [44]. The study results revealed that: (i) the doses decreased the mice body weights, while increasing the toxicant level in the brain; (ii) the object recognition test was disrupted by 3 mg and 10 mg doses but not by a 1 mg test set; and (iii) swimming had the ability to prevent the long-term memory impairment of the 3 mg group but not the 10 mg mice. In this study it was mentioned that arsenic exposure did not influence the total CREB expression. The swimming exercises prevented object recognition ability deterioration, which could be mediated by CREB and BDNF from the dorsal hippocampus. They concluded that the swimming exercises can exert the effect of removal arsenic and may activate some protection-based process.

Ramos-Chávez et al. [45] mentioned that the arsenic exposure has been linked with learning disabilities, memory impairment and lower intelligence coefficient. Authors’ utilized CD1 mouse models for tested the effects of gestational arsenic exposure, which leaded to many molecular events, such as unruly cysteine/glutamate transport, oxidative damage, and negative effects on learning and memory. Studies results showed that: ”(i) exposure to arsenic during gestation would be responsible for increasing oxidative stress thus up-regulating xCT (a component of the x_c_^−^ amino-acid transporter), (ii) 20 mg L^−1^ of arsenic failed to cause clear signs of toxicity in during gestation or before mating in CD1 mouse, (iii) day 90 males which were exposed during gestation while continuing to ingest water with arsenic showed memory deficits and an increase of expression in xCT and minor down-regulation of *N*-methyl-d-aspartate receptor subunit NR2B, but not subunit NR2A, suggesting a problem in synaptic efficiency, (iv) their results clearly indicate that gestational exposure to inorganic arsenic negatively impacts hippocampal *N*-methyl-d-aspartate (NMDA) receptor subunits expression” [45].

Kyaw Htet Aung et al. [46] mentioned that arsenic compounds are capable of inducing alterations in morphology and neuronal cell death, especially neurite outgrowth and the cyostekeleton of neurons. Arsenic exposure is responsible for alteration of the expression of nuclear factor cells, the decrease of nuclear factor transport to axonal outgrowth, as well as increasing perikaryal nuclear factor expression [46]. Aung’s team noticed that the exposure to sodium arsenite caused a significant increase in the gene expression of nuclear factor -L and nuclear factor -M proteins in neural cells while the neurite outgrowth suppression was also marked. This study strongly suggests that arsenic-induced neurite suppression leaded to nuclear factor dynamic alteration. They also observed that the microtubule gene expression, and also protein Tau, were significantly decreased due to exposure to sodium arsenite [46].

A recent study performed by Yachen Wang et al. [47] tested if arsenic exposure induced apoptosis in the hippocampus. The authors utilized 64 mice divided into four groups. Group 1 was the control group, thus receiving only water. For Groups 2–4 they were orally given arsenic trioxide (As_2_O_3_) at concentrations of 1, 2, and 4 ppm. The treatment lasted for 60 days. The results observed apoptosis and abnormal histopathological changes found in the hippocampus of mice who were exposed to arsenic [47]. The hippocampal expression of Bcl-2(B-cell lymphoma 2) gene and its protein were significantly lower than the control group’s results (*p* < 0.05). The expression ratio of Bax (BCL2 Associated X)/Bcl-2 and the expression of Bax gene and its protein were far higher in the arsenic groups compared to the control group. Even the activity of caspase-3 in the hippocampus was higher in the groups exposed to arsenic than control. This study concludes that sub-chronic dosages of arsenic disturbs the normal Bax/Bcl-2 pathways and is responsible for apoptosis in mice hippocampus. Arsenic-induced neurotoxicity may be partially explained by the induced apoptotic effect in the hippocampus [47].

Katharine E. Caldwell’s study [48] reported a possible molecular pathway of arsenic in the nervous system represented by the glucocorticoid system, which has an important role in many cellular functions including learning, memory, and mood disorders. From this study it was postulated that the disturbance of the glucocorticoid receptor (GR) pathway in the fetal brain and placenta are critical. Two critical periods represented by embryos, 14 and 18 days, were evaluated and the study was focused on 11β-hydroxysteroid dehydrogenases (11β-HSD), which exert a key function in the synthesis of glucocorticoid. They have identified that at the E14 time checkpoint arsenic exposure decreased in a significant manner the expression of enzyme 11β-HSD1, while the 11β-HSD2 enzyme level was increased. The changes continued into the E18 checkpoint, but mRNA levels stopped being altered significantly. In the arsenic-exposed condition, GR placental levels were decreased at E18. Sonia Ronchetti et al. [49] mentioned that arsenic can affect the anterior pituitary gland and uterus. The authors studied if low doses of in vivo administration through drinking water would display xenoestreogenic effects in the uterus and anterior pituitary gland of ovariectomized rats. Arsenic significantly affects the anterior pituitary by exerting strong xenoestreogenic effects [49].

## 3. Perturbance of Neuropsychiatric Treatments during Arsenic Exposure

Arsenic exposure, particularly the chronic type, can lead to poisoning with manifestations presenting in multiple organ systems. It is very important to prove that the nervous system damages induced by arsenic are reversible.

However, acute psychosis is not a commonly described manifestation of arsenic exposure. Wu et al. [50] published in their work a study about acute psychosis induced by arsenic exposure. According to Wu et al. patients presented a severe symptomatology which includes obsessive compulsive symptoms, psychosis, and hallucinations. By treating such patients with a combination of antipsychotic and antidepressant drugs a significant improvement in obsessive-compulsive and acute psychosis symptoms was observed. Based on the research results the authors conclude that following arsenic poisoning patients can develop several atypical symptoms, including acute psychosis. The treatment of patient was represented by combinatorial therapies with antidepressants and neuroleptics but is not clear the molecular mechanism by which these drugs are able to combat psychosis symptoms.

A very interesting paper was published by Christina R. Tyler et al. [51] about the reduced perinatal symptoms of depression during fetal exposure of arsenic during fluoxetine treatment. In their study authors, assessed the effects of fluoxetine, a strong antidepressant with a selective serotonin reuptake inhibitor role, on adult animals exposed to arsenic during development. Perinatal arsenic exposure induced depressive-like symptoms in a mild learned helplessness task and in the forced swim task after acute exposure to 2,4,5-trimethylthiazoline (TMT). The results showed that: (i) the chronic fluoxetine treatment prevented depression behaviours in case of arsenic-exposed; (ii) reduced the arsenic-induced blunted stress responses; (iii) chronic fluoxetine treatment reversed deficits in adult hippocampal neurogenesis after arsenic exposed. This study demonstrates that damage induced by perinatal arsenic exposure is reversible with chronic fluoxetine treatment.

Additionally, it was proven that fluoxetine is a powerful antidepressant during neurogenesis in the human adult hippocampus [52] by acting on serotonin receptor 5-TH1A and also the sigma1 receptor (σ1R), but many molecular aspects of these processes are still unclear. Avram et al. [52] published a review about possible antidepressants inducing enhanced effects on neurogenesis on 5-TH1A and also the sigma1 receptor. Among these antidepressants, in our previous study we mentioned: escitalopram, sertraline, paroxetine, or duloxetine. In our opinion if the chronic treatment with fluoxetine can reduce the toxic effect of arsenic in the body, we suggest that future studies involve the chronic treatment with antidepressants escitalopram, sertraline, and paroxetine, and antipsychotics, like piperazine, should be very important for treatment during arsenic exposure.

## 4. Bioinformatics and Cheminformatics Tools Applied to Study Arsenic Toxicity in the Brain

In silico approaches of arsenic toxicity represent a new way to identify its neutralization in the human body. Regarding this aim, the friendly bioinformatics (various databases, predictive ADMET—absorption, distribution, metabolism excretion and toxicity) and cheminformatics (quantitative chemical structure-biological activity relationship (QSAR), molecular docking, high-throughput screening) tools were clearly recognized as very useful tools. QSAR is a very useful tool in medical chemistry, allowing the investigation of the interactions between chemical compounds (drugs, pro-drugs, various ligands, pollutants) and their macromolecular targets (e.g., enzymes, membrane receptors) [53,54,55].

Basically, QSAR methods considered that the biological activity is induced by the molecular structure and every change in molecular structure leads to a modification of these properties [56,57,58,59,60,61]. There are few differences among QSAR methods and these consisted in the way they describe the structural properties of compounds and in the quantitative relationships found between these properties and activities [21,62]. In QSAR, the molecular descriptors could be classified in: (i) atom and bound counts, molecular weight, and the sum of atomic properties; (ii) molecular fragment counts; (iii) topological descriptors; (iv) atomic coordinates or energy grid descriptors; and (v) the combination of atomic coordinates and sampling of conformations. Additionally, QSAR methods consider: (i) different types of probe atoms; (ii) different force fields; and (iii) different manners of calculating the electronic interactions [53].

In QSAR methods, the appropriate selection of molecular descriptors assures the QSAR model’s prediction power. Usually, the biological activity of compounds can be correlated with large sets of molecular descriptors, such as: (i) steric (subdivided van der Waals surface and volume, subdivided solvent accessible surface and volume); (ii) atom and bond counts (hydrophobic/polar, donor/acceptor atoms, rigid and rotatable bonds); (iii) hydrophobicity and solubility; and (iv) electronic descriptors (subdivided potential energy, molar refractivity, dipole moment, energies of frontier molecular orbital) [63].

Presently, the predictive ADME-Tox (absorption, distribution, metabolism, and excretion toxicity) for compounds are an important challenge. Among bioinformatics tools used in predictive ADME-Tox, there are few databases (TOXNET, Drugbank, admetSAR, IDA2PM, etc.) [64,65,66] which are very useful and freely accessible. There are also molecular simulation software packages (Discovery Studio, Volsurf) [27,67] used to predict the toxicological and structural features of chemical compounds. Below, we briefly present some of them.

The National Library of Medicine (NLM) portal provides reliable information on toxicology, hazardous chemicals, environmental health, and toxic announcements [64]. TOXNET (Toxicology Data Network), belonging to the NLM portal, is a free database system with information on toxicology, environmental health, chemical nomenclature, poisoning, risk assessment and regulations, and occupational safety and health. It is produced by the Specialized Information Services Division of the US. From this database we selected a few useful links and, very importantly, the field covered by these (Table 2).

Another important bioinformatics database is DrugBank [65]. DrugBank is a wide-range database that contains information on drugs and drug targets. As a bioinformatics and cheminformatics source, DrugBank collects detailed information regarding drug features (i.e., chemical, pharmacological, and pharmaceutical) with comprehensive drug target information (i.e., sequence, structure, and pathway). The database contains over 8000 drug entries including chemicals and additionally, 4270 non-redundant protein (i.e., drug target/enzyme/transporter/carrier) sequences are linked to these drug entries [68].

We search arsenic and its derivatives in DrugBank and we mentioned that there is just one arsenic derivative, namely arsenic trioxide, a very important compound in tumor treatment (Table 3). We mentioned that the important experimental ADMET features of arsenic trioxide are not elucidated yet.

Based on our expertise in bioinformatics and cheminformatics, here we accessed the bioinformatics tools Accelerys Discovery Studio (DS) [27] and the online ADME-Tox database [69], in order to predict the few ADME-Tox features of arsenic and its derivative arsenic trioxide (Table 4).

**Figure 1:**
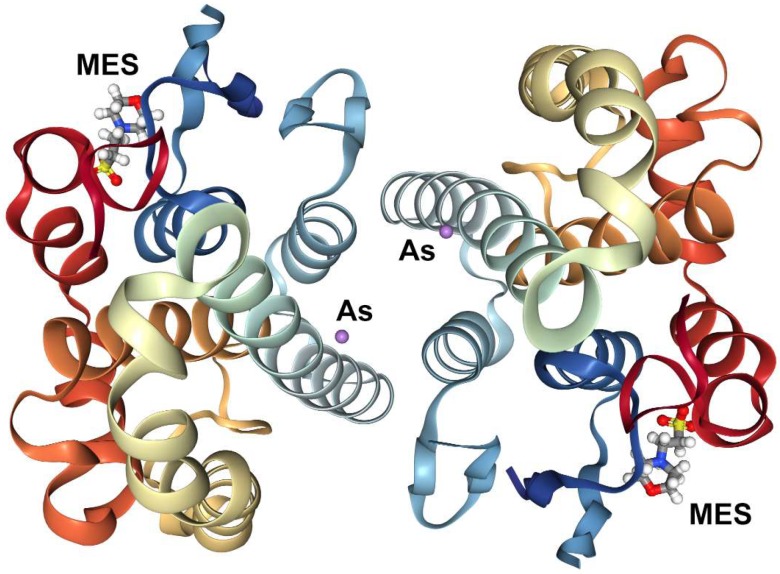
Crystal structure of human glutathione transferase pi complexed with a metalloid in the absence of glutathione, PDB ID: 5DAK: molecule: glutathione S-transferase P (chain: A, B), heteroatoms CSO (S-hydroxycysteine), MES (2-(n-morpholino)-ethanesulfonic acid) and As (arsenic).

Another important database that contains the structural and functional information of proteins in complex with their ligands is Protein Data Bank [70]. This database contains information regarding protein and nucleic acids obtained from X-ray diffraction, nuclear magnetic resonance, cryo-electron microscopy collected by biologists and biochemist from around the world, being freely available over the Internet [70]. We search the molecular complex of arsenic and we found PDB code 5DAK, posted by Parker et al. [71] about the crystal structure of human glutathione transferase pi complexes with a metalloid in the absence of glutathione (Figure 1).

In 2014, Canaval et al. [72] published a study about hybrid ab initio quantum mechanics and molecular mechanical simulations applied to investigate the hydrolysis process of arsenic(III), ultimately leading to arsenous acid (H_3_AsO_3_). In this study are discussed: (i) the geometrical properties of H-bonds involved in each of the three protons transfer; and (ii) subsequent proton bounding reactions. For results interpretation Laguerre tessellation analysis has been employed in order to estimate the molecular volume of H_3_AsO_3_. As novelty of this study, the authors performed Fourier transform infrared (FT-IR) spectroscopy measurements, which have been compared to power simulation spectra. The study results, represented by simulation findings, as well as results from computational spectroscopic calculations utilizing the PT2-VSCF methodology, fitting the experimental FT-IR data well.

Another interesting paper regarding the critical molecular descriptors involved in arsenic toxicity was published by Roy et al. [73]. In this study the following were considered as critical molecular descriptors: atomic number (Z) and electrophilicity index (omega) in order to explain toxicity of various alkali and arsenic ions. The results studied were mentioned as critical molecular descriptors of toxicity: (i) the global electrophilicity (omega), (ii) number of non-hydrogenic atoms (N (NH)); (iii) philicity (omega); and (iv) the atomic charge (Q) on the arsenic. Additionally, by applying the regression models from the training sets, the toxicity of some unknown arsenic derivatives is predicted.

A helpful use of arsenic was mentioned by Zhang et al. [74]. In this paper it was mentioned that arsenic trioxide presented a great clinical success in the treatment of acute promyelocytic leukemia (APL). In this paper the authors reported that “a “one-pot” method to develop arsenic-based nanodrugs by in situ coating the as-prepared arsenic nanocomplexes with porous silicashells”. Here it was mentioned that, in accord with these unique features, the nanodrugs are able to strongly inhibit the growth of solid tumors. These effects are very important; in addition, these principal effects happened without adverse side effects. The authors, based on their results, suggested that the arsenic-based nanodrugs generated by this facile synthetic route may be a powerful and alternative strategy for solid tumor therapy.

A very interesting paper was published by Wei Kheng Teoh et al. [75], in order to establish the description of *Thiomonas delicata* arsenite oxidase identified in *Escherichia coli* by using bioinformatics and cheminformatics. This study represents the first characterization of arsenite oxidase found in *Thiomonas* genus. It was mentioned that arsenite oxidase presents a real potential to be used for biosensors and bioremediation applications in acidic environments. For the performed homology model of arsenite oxidase from *T. delicate*, the authors used a SWISS-MODEL workspace, and used as template the arsenite oxidase crystal structure isolated from *A. faecalis* (PDB: 1G8K chains A and B). The structural superimposition and graphical representation of the structure model was prepared in UCSF CHIMERA software.

The homology results showed that: (i) the reliability of the model was supported by its high sequence identity of 64% and with QMEAN4 score of −2.16; (ii) a homology comparison of the model and template showed a high similarity in overall fold and active site residues; and (iii) electrostatic charges on both enzymes revealed that the surfaces of arsenite oxidase in *T. delicata* are rich with positively-charged residues, while arsenite oxidase in *A. faecalis* has more negatively-charged surface residues.

An interesting study was performed by Tsai et al. [76] in order to employ molecular dynamics for elucidation of structural/dynamic features of arsenic in a lipid bilayer. In this study, it was considered that the cell membranes are composed mainly of phospholipids which are, in turn, composed of five major chemical elements: carbon, hydrogen, nitrogen, oxygen, and phosphorus. Tsai et al. studied the arsenated-lipid bilayer interactions by simulating the arsenic-lipid interaction, 1-palmitoyl-2-oleoyl-*sn*-glycero-3-arsenocholine (POAC) and arsenic-lipid 1-palmitoyl-2-oleoyl-*sn*-glycero-3-phosphocholine, (POPC). The structural and dynamical properties of these two complexes was mentioned, with emphasis on the differences among them. The results studied reveal that: POAC lipid bilayers present different structural and dynamical features from those of native POPC lipid bilayers; (ii) “compact structure of POAC lipid bilayers is due to the fact that more inter-lipid salt bridges are formed with arsenate-choline compared to the phosphate-choline of POPC lipid bilayers”; and (iii) POAC and POPC lipid bilayers would have different biological implications.

Molecular dynamic protocols were used in CHARMM-GUI, developed by Jo et al. [77]. The initial configurations of the POAC and POPC systems were prepared. Water molecules were modeled using the TIP3 model [78,79].

Another study performed by Altaf Hussain Pandith et al. [80] reveal the importance of two molecular descriptors in the toxicity of aliphatic compounds from *Tetrahymena pyriformis*. A series of important molecular descriptors, such as: orbital energy (LUMO energy, HOMO energy), ionization energy, chemical potential, electron affinity, electronegativity, philicity, and electrophilicity of a series of aliphatic compounds are calculated. In this study were involved a large series of molecules, such as: (i) polyaromatic hydrocarbons (e.g., polychlorinated biphenyls (PCBs)), polychlorinated dibenzofurans (PCDFs), polychlorinated dibenzo-*p*-dioxins (PCDDs), and chlorophenols (CP), as well as arsenic derivatives. A wide number of QSAR models were presented and the statistic parameters R^2^ correlation, Fisher F, and unexplained variance RSS, recorded significant values.

The results studied revealed that:

(i) For various chemical structures the critical molecular features for structure toxicity is different;
(ii) The chemical structure constructed based on the electrophilicity index (ω) and log P (R^2^adj = 0.965 for acceptors, 0.888 for donors) is better than the model based on eLUMO and log P (R^2^ = 0.963 for acceptors, 0.842 for donors); and
(iii) The QSAR model can improve molecules’ toxicity predictability, and can be developed by taking into account the electrophilic property in addition to molecule hydrophobicity.

Recently, Hui Dong et al. [81] reported new small molecules as possible inhibitors of human As(III) S-adenosylmethionine methyltransferase (AS3MT) by molecular docking. It was mentioned that, in humans, arsenic is primarily metabolized by the enzyme As(III) SAM methyltransferase (hAS3MT), a member of the large superfamily of SAM methyltransferases (SAM MTs) [82,83,84] knowing that methylation is important for human biochemical processes (Table 5).

Considering it, the biochemical events are: “AS3MT biotransforms of inorganic arsenic (As(III)) into trivalent methylated species methylarsenite (MAs(III)) and dimethylarsenite (DMAs(III)) using SAM as the methyl donor”. The major aim of this study was to report new hAS3MT ligands, able to modulate enzyme activity. In this study authors identified 10 compounds that inhibit hAS3MT with IC50 values varies from 30 μM to 50 μM. None of them inhibit the binding of either As(III) or MAs(III), and they do not inhibit catachol o-methyltransferase (COMT), a nonarsenic SAM MT. In silico methods, namely molecular docking, were used in this study. By using the PDB database, CmArsM (4FS8) and COMT (3BWM) structures were prepared for docking analysis, adding Gasteiger charges.

**Figure 2:**
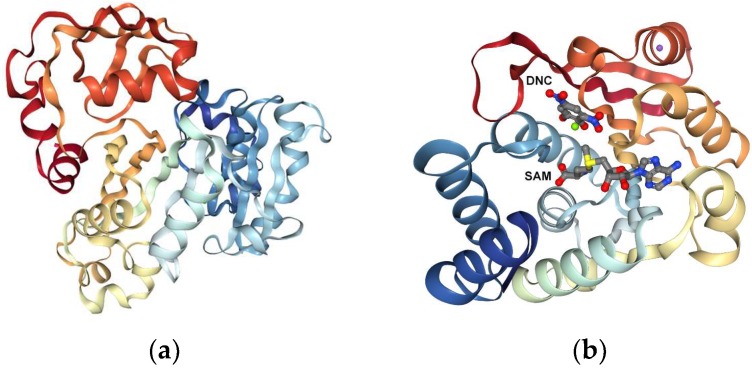
(**a**) The structure of an As(III) S-adenosylmethionine methyltransferase: insights into the mechanism of arsenic biotransformation (4FS8). (**b**) Crystal structure of human catechol O-methyltransferase chain: A with bound SAM (S-adenosyl methionine) and DNC (PDB code: 3BWM).

The study results revealed that all 10 ligands inhibited As(III) methylation, but only five inhibit MAs(III) methylation, indicating differences in their mechanism of action. The authors proposed that AS3MT suffers different conformational changes during the two methylation steps that may account for the differential action of the inhibitors (Figure 2).

>3BWM:A|PDBID|CHAIN|SEQUENCE
GDTKEQRILNHVLQHAEPGNAQSVLEAIDTYCEQKEWAMNVGDKKGKIVDAVIQEHQPSVLLELGAYCGYSAVRMARLLSPGARLITIEINPDCAAITQRMVDFAGVKDKVTLVVGASQDIIPQLKKKYDVDTLDMVFLDHWKDRYLPDTLLLEECGLLRKGTVLLADNVICPGAPDFLAHVRGSSCFECTHYQSFLEYREVVDGLEKAIYKGP
>4FS8:A|PDBID|CHAIN|SEQUENCE
MPCSCASGCQKSKNGGSTPSIRDHVADYYGKTLQSSADLKTSACKLAAAVPESHRKILADIADEVLEKFYGCGSTLPADGSLEGATVLDLGCGTGRDVYLASKLVGEHGKVIGVDMLDNQLEVARKYVEYHAEKFFGSPSRSNVRFLKGFIENLATAEPEGVPDSSVDIVISNCVCNLSTNKLALFKEIHRVLRDGGELYFSDVYADRRLSEAAQQDPILYGECLGGALYLEDFRRLVAEAGFRDVRLVSVGPVDVSDPQLRKLVPDVQFYSCTFRCFKVATLEATREDYGQSATYLGGIGEEFKLDRFFTFPREKPVRVDRNTAEIIRHSRLHQWFSVSAEQQHMGLFKANDSYALLHAPLSMQVEQLVSGAAALEHHHHHH

## 5. Arsenic Reduced Toxicity and Arsenic Nanoparticles Used as Delivery System into the Human Body

Recently, basic QSAR was used for predicted molecular features of nanoparticles, usually renamed as nano-QSAR. Classical QSAR models, as convertors of chemical information, show some disadvantages in predicting the features of nanomaterials, especially their toxicity [60,61]. During the last decades high interest in the development of “theranostic pharmaceuticals” was observed, allowing for the initial diagnostic and probing of a specific pharmacological target. Regarding these applications, arsenic was used in the form of arsenic radioisotopes, with considerable interest in the field of nuclear medicine [85].

In nuclear medicine, “theranostics” are chemical compounds (radionuclides) with similar structural features, but different radioactive disintegration properties, such as: (i) one with a positron- or gamma-emitting nuclide for positron-emission tomography (PET); (ii) single-photon emission computed tomography diagnostic imaging; and (iii) one with an electron-, alpha-, or Auger-emitting nuclide [85]. From many radioisotopes, radioisotopes of arsenic offer an alternative radionuclide-based theranostic system. Arsenic has unique chemical and biochemical properties that make it notable for novel diagnostic or therapeutic radiopharmaceuticals [85].

Even if arsenic trioxide has been approved for the treatment of refractory acute promyelocytic leukemia it was mentioned that it is difficult to apply to other cancers, such as solid tumors, in part because of the rapid renal clearance and dose-limiting toxicity [74].

Zongjun Zhang et al. developed a new arsenic-based nano-drug using porous silica shells by using a “one-pot” synthesis method. The authors mention that “this process can be easily reproduced and scaled up because no complicated synthesis and purification steps are involved. This core–shell embedding method endows nanodrugs with high loading capacity (57.9 wt%) and a prolonged pH-responsive releasing profile, which is crucial to increase the drug concentration at tumor sites and improve the drug efficacy” [74]. Nickel(II) arsenite nanocomplexes (NiAsOx) are prepared as ATO-based prodrugs in microemulsion prior to further silica shell coating (NiAsOx@SiO_2_). The nanocomposites are then modified with zwitterionic ligands (denoted as NiAsOx@SiO_2_-ZW), which render colloid stability over biological conditions and increased half-life in blood

Animal studies indicated that NiAsOx@SiO_2_-ZW nanocomposites effectively suppressed the tumor growth compared with free ATO. Regarding the cytotoxicity of arsenic-loaded nanoparticles it was mentioned that “after 48 h incubation with these three cell lines (Fig. 5), both NiAsOx@SiO_2_ (IC50 = 0.58, 2.26, 38.08 μM, respectively) and NiAsOx@SiO_2_-ZW (IC50 = 2.73, 9.52, 42.10 μM, respectively) showed better arsenic bioavailability and significantly higher cytotoxicity than free ATO (IC50 = 13.79, 25.42, 47.52 μM, respectively)”. Regarding the in vivo behavior of nanodrugs, the results suggested that NiAsOx@ SiO_2_-ZW may be more effective for the accumulation in solid tumor sites compared to NiAsOx@SiO_2_ through the EPR effect. In vivo therapy showed that the treatment of NiAsOx@SiO_2_-ZW effectively suppressed the tumor growth, resulting in a mean tumor volume. It should be noted that the NiAsOx@SiO_2_-ZW was more effective in suppressing tumor growth than free ATO.

Another important part to reducing arsenic toxicity in the body is represented by the elimination of it in water. One of the simplest and cheapest processes for arsenic removal from water, also being considered a natural process, is the process in which water is passed over a sand filter. The affinity of arsenic for iron and manganese ions explained the arsenic removal present in underground water by simply passing over a filterable bed formed by sand, a process determined by the presence of significant concentrations of iron and manganese ions in this type of water. The process of arsenic removal consists in its retaining due to the presence of iron hydroxo-oxides or manganese oxides. In the aqueous solution arsenic is found either in the arsenate As(III) form or arsenite As(V) form.

The mechanism on which it is based, the process of arsenic removal from water through adsorption predominantly depends on the nature of the adsorbent material. Selective adsorption using biological materials, mineral oxides, activated carbon, and industrial waste or polymeric resins has generated enthusiasm leading to good results [86,87]. Even if the surface chemistry of carbon depends on the activation conditions, temperature, by the way of refining the structure of pores (micro- and mesopores) formed, which can lead to specific surfaces around 2000 m^2^ g^−1^, this does not mean that they are the determinant factor of activated carbon’s high adsorption capacity [88,89,90].

Arsenic species present in water limit the performance of many adsorbent materials, most of which are much more suitable for removing As(V) than As(III) [91]. Since arsenic species present a good affinity for iron, iron oxides/hydroxides have been extensively studied in order to understand arsenic adsorption behavior. Traditional methods used to remove arsenic from water are generally expensive and present a major inconvenience of generating by-products. Thus, attention is granted to implement new methods of reducing/recovering arsenic from aqueous solutions by developing clean technologies.

In recent years, implementing at large scale the arsenic removal from aqueous solutions by adsorption, it has been necessary to develop advanced adsorbent materials with applications in this process. In order to improve the adsorption properties of materials, new methods of chemical modification of the inorganic or organic adsorbents were developed by functionalization of such materials with different extractants dissolved in different solvents.

Presently, the used methods are: wet method (the diluted extractants dissolved in different solvents is kept in contact with the solid support, the liquid phase being absorbed by the support), dry method (the extractant is diluted with the solvent, put in contact with the solid support, after which the solvent is removed through slow evaporation under vacuum), modifier addition method (this method is an hybrid between the dry and the wet method), and the column dynamic method, which presents the advantages of a short time of functionalization correlated with an increased efficiency of the adsorption process [92,93,94,95,96,97].

In order to apply these methods, the extractants should be liquid or kept in a liquid form by adding a proper solvent; the extractant and the solvent must have a minimum solubility; the support must be prepared for the impregnation and the functionalization method does not have to modify the extractants or the support properties [98].

Frequently used as solid supports are macroporous polymeric resins with a rigid three-dimensional structure (XAD-type resins), appropriate to incorporate higher quantities of extractants due to the proper specific surface area, great mechanical resistance, and low solvent swelling during the functionalization process. These amberlite resins are used at the large-scale, being produced as: styrene-divinyl benzene aromatic copolymer (XAD-2, XAD-4, etc.), aliphatic methacrylate copolymer (XAD-7, XAD-8, etc.), and divinyl benzene aromatic copolymer (XAD-12, XAD-16, etc.) [92].

## 6. Conclusions and Perspective

Serious health problems caused by arsenic toxicity, it mechanisms of neurotoxicity, and how the treatment or other methods could be able to reduce the concentration of arsenic in the brain are unclear. In this paper, we presented the possibility of a new approach of arsenic removal.

Exposure of high quantities of arsenic, lead, and manganese has negative effects affecting the whole body. It was proved that exposure to arsenic can induce genotoxicity and cancer, which affect the liver, skin, kidneys, or brain. Arsenic’s inorganic form is linked with impairing memory and its effect was studied on spatial memory, proving that long-term exposure affects brain performance. Arsenic-induced toxicity can be partially explained considering the induced apoptotic effect in the hippocampus. Experiments performed during the last decade proved that acute psychosis can be induced by arsenic exposure, with severe symptomatology: hallucinations, disorganized thinking, and obsessive-compulsive symptoms. By treating patients with a combination of antipsychotic and antidepressant drugs a significant improvement of the acute psychosis and obsessive-compulsive symptoms was observed.

In silico approaches of arsenic toxicity represent a new way to identify its neutralization in the human body. Regarding this aim, bioinformatics (various database, predictive ADMET) and cheminformatics (quantitative chemical structure-biological activity relationship (QSAR), molecular docking, high-throughput screening) tools were clearly recognized as very useful tools.

A different approach is represented by the usage of arsenic trioxide as an active drug for the treatment of promyelocytic leukemia, where the nanodrugs can inhibit the growth of solid tumors. These effects are very important, besides these principal effects happening without adverse side effects. Even if arsenic trioxide has been approved for the treatment of refractory acute promyelocytic leukemia it was mentioned that it is difficult to apply to other cancers, such as solid tumors, in part because of the rapid renal clearance and dose-limiting toxicity. For future usage of arsenic trioxide as an antitumoral drug it is important to develop arsenic-based nanodrugs by in situ coating of arsenic nanocomplexes with porous silica shells or with some other drug carriers.

Water is an essential element for life and for many for natural processes; our existence and our economic activities are totally dependent on this precious resource. Moreover, globally, water represents a limited resource. Despite the numerous efforts and financial resources invested to find solutions, millions of people around the world are predisposed to daily consumption of arsenic-polluted water.

We used iron-based oxide materials synthesized by combustion or calcination, as well as unconventional materials (sludge obtained from remediation of wastewater with a high content of metallic oxides, especially iron), but also some modern materials (commercially- or chemically-modified synthetic polymers) in the process of arsenic removal from water. At the same time, the possibility of closing the cycle by inerting the waste, resulting in arsenic immobilization into vitreous matrices in order to obtain useful products, was presented.

## Figures and Tables

**Table 1:** Models of metals exposure and BSID-III scores regarding cognitive and fine motor activities collected from children living in Sirajdikhan and Pabna, Bangladesh, adapted after [24].

Exposures	Cognitive Score				Fine Motor Score			
	Sirajdikhan, *n* = 239 Children		Pabna, *n* = 286 Children		Sirajdikhan, *n* = 239 Children		Pabna, *n* = 285 Children	
	Statistical Values							
	β (SE)	*p*-Value	β (SE)	*p*-Value	β (SE)	*p*-Value	β (SE)	*p*-Value
Water and Arsenic	−0.002 (0.02)	0.93	−0.06 (0.03)	0.05	−0.05 (0.03)	0.09	0.02 (0.03)	0.48
Water and Manganese (Mn)	0.02 (0.02)	0.35	−0.06 (0.07)	0.33	−0.04 (0.03)	0.21	0.85 (0.39)	0.03
Water and Manganese (Mn2)	-	-	-	-	-	-	−0.08 (0.03)	0.02
Blood and Lead metal (Pb)	−0.17 (0.09)	0.05	0.02 (0.12)	0.87	0.07 (0.11)	0.50	−0.07 (0.11)	0.50

**Table 2:** The databases able to be access TOXINET, and their covered fields [64].

**ChemIDplus**	Chemical names, formulas, structures
**CCRIS**	Carcinogenicity, mutagenicity
**CPDB**	Cancer testing
**CTD**	Toxicogenomics information
**GENE-TOX**	Mutagenicity test data
**IRIS**	Human health risk assessment
**ITER**	Risk information
**TOXLINE**	Toxicology journal literature
**DART**	Reproductive toxicology journal literature
**Haz-Map**	Occupational health
**HSDB**	Health effects, toxicity, regulations
**TOXMAP**	Interactive U.S. maps of chemical releases

**Table 3:** Structural, functional, and pharmacokinetic data of arsenic trioxide extracted from the DrugBank database [65].

**ID arsenic Trioxide**	DB01169
**Chemical Formula**	As_2_O_3_
**SMILES**	O=[As]O[As]=O
**Therapeutic Indication**	“For induction of remission and consolidation in patients with acute promyelocytic leukemia (APL), and whose APL is characterized by the presence of the t(15;17) translocation or PML/RAR-alpha gene expression”
**Pharmacodynamics**	“Arsenic Trioxide is indicated for induction of remission and consolidation in patients with acute promyelocytic leukemia (APL) who are refractory to, or have relapsed from, retinoid and anthracycline chemotherapy.”
**Absorption**	Not available
**Volume of Distribution**	Not available
**Protein Binding**	75% bound
**Route of Elimination**	Trivalent arsenic is mostly methylated in humans and excreted in urine
**Half Life**	Not Available
**Clearance**	Not Available
**Toxicity**	“Symptoms of overdose include convulsions, muscle weakness and confusion”

**Table 4:** Predictive ADME-Tox (absorption, distribution, metabolism, and excretion and toxicity) features of arsenic trioxide and arsenic [27,69].

Arsenic Trioxide		
ADME-Tox	Features	Unit
**Absorption**	Water solubility (log S)	0.84 (log mol/L)
	Intestinal absorption	100%
**Distribution**	Volume of distribution (human)	−0.48 log (L/Kg)
	Blood-brain Barrier Permeability (BBB)	−0.361(log BB)
	Central Nervous System permeability (CNS)	−2.70 (log PS)
	Fraction unbounded of plasma proteins	75%
**Metabolism**	CYP2D6 substrate/inhibitor	NO
**Excretion**	Total clearance	1.064 (log mL/min/kg)
**Toxicity**	AMES (mutagenic feature)	NO
	Hepatotoxicity	NO
	Skin permeability	NO
	Max. tolerated dose (human)	1.125 (log mg/kg/day)
	Oral rat acute toxicity (LD50)	2.331 (mol/kg)
**Arsenic**		
**Distribution**	Blood-brain Barrier Permeability (BBB)	0.003 (log BB)
	Central Nervous System permeability (CNS)	−2.30 (log PS)
**Toxicity**	AMES (mutagenic mutagenic)	NO
	Hepatotoxicity	NO
	Skin permeability	NO
	Max. tolerated dose (human)	1.18 (log mg/kg/day)
	Oral rat acute toxicity (LD50)	2.21(mol/kg)

**Table 5:** Comparison of binding properties of 10 small molecule inhibitors to AS3MT calculated using Autodock Vina33 [81].

Compound	hAS3MT Binding Affinity (kcal/mol)	hAS3MT Kd (μM)	Catachol O-Methyltransferase (COMT) Binding Affinity (kcal/mol)	Catachol O-Methyltransferase COMT Kd (μM)
TPI-1	−7.3	4.5	−4.3	704
TPI-2	−8.4	0.7	−5.6	79
TPI-3	−9.3	0.2	−4.6	424
TPI-4	−8.0	1.3	−3.6	229
TPI-5	−9.4	0.1	−1.4	9410
TPI-6	−7.2	5.3	−4.7	358
TPI-7	−8.5	0.6	−5.0	216
TPI-8	−9.2	0.2	−2.2	2440
TPI-9	−8.8	0.5	−0.7	306,800
TPI-10	−9.0	0.3	−5.5	93

## References

[1] Andrade V.M., Batoréu M., Aschner M., Marreilha dos Santos A. (2015). Lead, arsenic and manganese metal mixture exposures: Focus on biomarkers of effect. Biol. Trace Element Res..

[2] United States Environmental Protection Agency. https://www.epa.gov/.

[3] Agency for Toxic Substances and Disease Registry (ATSDR). https://www.atsdr.cdc.gov/.

[4] Siddiqui S.I., Chaudhry S.A. (2017). Iron oxide and its modified forms as an adsorbent for arsenic removal: A comprehensive recent advancement. Process Saf. Environ. Prot..

[5] Kao A.C., Chu Y.J., Hsu F.L., Liao V.H.C. (2013). Removal of arsenic from groundwater by using a native isolated arsenite-oxidizing bacterium. J. Contam. Hydrol..

[6] Bowell R.J., Alpers C.N., Jamieson H.E., Nordstrom D.K., Majzlan J. (2014). The Environmental Geochemistry of Arsenic-An Overvi. Rev. Mineral. Geochem..

[7] McIntyre D.O., Linton T.K. (2011). Arsenic. Fish Physiol..

[8] Smedley P.L. (1996). Arsenic in rural groundwater in Ghana. J. Afr. Earth Sci..

[9] Mohan D., Pittman C.U. (2007). Arsenic removal from water/wastewater using adsorbents—A critical review. J. Hazard. Mater..

[10] Qu D.W.J., Hou D., Luan Z., Fan B., Zhao C. (2009). Experimental study of arsenic removal by direct contact membrane distillation. J. Hazard. Mater..

[11] Kundu S., Gupta A.K. (2007). As(III) removal from aqueous medium in fixed bed using iron oxide-coated cement (IOCC): Experimental and modeling studies. Chem. Eng. J..

[12] Avram S., Maria M., Mihailescu D., Duda-Seiman D., Duda-Seiman C. (2013). Advanced QSAR Methods Evaluated Polycyclic Aromatic Compounds Duality as Drugs and Inductors in Psychiatric Disorders. Curr. Org. Chem..

[13] Perera F.P., Wheelock K., Wang Y., Tang D., Margolis A.E., Badia G., Cowell W., Miller R.L., Rauh V., Wang S. (2018). Combined effects of prenatal exposure to polycyclic aromatic hydrocarbons and material hardship on child ADHD behavior problems. Environ. Res..

[14] Fluegge K., Fluegge K. (2018). Environmental factors influencing the link between childhood ADHD and risk of adult coronary artery disease. Med. Hypotheses.

[15] Goodrich A.J., Volk H., Tancredi D.J., McConnell R., Lurmann F.W., Hansen R.L., Schmidt R.J. (2018). Joint effects of prenatal air pollutant exposure and maternal folic acid supplementation on risk of autism spectrum disorder. Autism Res..

[16] Attademo L., Bernardini F., Garinella R., Compton M.T. (2017). Environmental pollution and risk of psychotic disorders: A review of the science to date. Schizophr. Res..

[17] Hudecova A.M., Hansen K., Mandal S., Berntsen H.F., Khezri A., Bale T.L., Fraser T.W.K., Zimmer K.E., Ropstad E. (2018). A human exposure based mixture of persistent organic pollutants affects the stress response in female mice and their offspring. Chemosphere.

[18] Castro B.B., Silva C., Macário I.P.E., Oliveira B., Gonçalves F., Pereira J.L. (2018). Feeding inhibition in Corbicula fluminea (O.F. Muller, 1774) as an effect criterion to pollutant exposure: Perspectives for ecotoxicity screening and refinement of chemical control. Aquat. Toxicol..

[19] De Vizcaya-Ruiza A., Barbier O., Ruiz-Ramos R., Cebrian M.E. (2009). Biomarkers of oxidative stress and damage in human populations exposed to arsenic. Mut. Res..

[20] Chang C.-Y., Guo H.-R., Tsai W.-C., Yang K.-L., Lin L.-C., Cheng T.-J., Chuu J.-J. (2015). Subchronic Arsenic Exposure Induces Anxiety-Like Behaviors in Normal Mice and Enhances Depression-Like Behaviors in the Chemically InducedChemically-induced Mouse Model of Depression. BioMed Res. Int..

[21] Duda-Seiman D.M., Avram S., Mancas S., Careja V., Duda-Seiman C., Putz M.V., Ciubotariu D. (2011). MTD-CoMSIA modelling of HMG-CoA reductase inhibitors. J. Serbian Chem. Soc..

[22] Hill D.S., Cabrera R., Wallis Schultz D., Zhu H., Lu W., Finnell R.H., Wlodarczyk B.J. (2015). Autism-Like Behavior and Epigenetic Changes Associated with Autism as Consequences of In Utero Exposure to Environmental Pollutants in a Mouse Model. Behav. Neurol..

[23] Dickerson A.S., Mohammad H., Rahbar I., Han A.V., Bakian D.A., Bilder R.A., Harrington S., Pettygrove M., Durkin R.S., Kirby M.S. (2015). Autism spectrum disorder prevalence and proximity to industrial facilities releasing arsenic, lead or mercury. Sci. Total Environ..

[24] Rodríguez-Barranco M.G.F., Hernández A.F., Alguacil J., Lorca A., Mendoza R., Gómez I., Molina-Villalba I., González-Alzaga B., Aguilar-Garduño C., Rohlman D.S. (2016). Postnatal arsenic exposure and attention impairment in school children. Cortex.

[25] Bode M.M.D.D., Mettelman B.B., Gross S.J. (2014). Predictive validity of the Bayley, Third Edition at 2 years for intelligence quotient at 4 years in preterm infants. J. Dev. Behav. Pediatr..

[26] Von Stackelberg K.G.E., Chu T., Henn B.C. (2015). Exposure to Mixtures of Metals and Neurodevelopmental Outcomes: A Review. Risk Anal..

[27] Dassault Systemes—Biovia. http://www.3dsbiovia.com/products/collaborative-science/biovia-discovery-studio/.

[28] Chou C.T.L.W., Kong Z.L., Chen S.Y., Hwang D.F. (2013). Taurine prevented cell cycle arrest and restored neurotrophic gene expression in arsenite-treated SH-SY5Y cells. Amino Acids.

[29] Ademuyiwa O.U.R., Rotimi S.O., Abama E., Okediran B.S., Dosumu O.A., Onunkwor B.O. (2007). Erythrocyte acetylcholinesterase activity as a surrogate indicator of lead-induced neurotoxicity in occupational lead exposure in Abeokuta, Nigeria. Environ. Toxicol. Pharmacol..

[30] Rosemberg D.B., da Rocha R.F., Rico E.P., Zanotto-Filho A., Dias R.D., Bogo M.R., Bonan C.D., Moreira J.C.F., Klamt F., Souza D.O. (2010). Taurine prevents enhancement of acetylcholinesterase activity induced by acute ethanol exposure and decreases the level of markers of oxidative stress in zebrafish brain. Neuroscience.

[31] Santos D.M.D., Andrade V., Batoréu M.C., Aschner M., Marreilha dos Santos A.P. (2012). The inhibitory effect of manganese on acetylcholinesterase activity enhances oxidative stress and neuroinflammation in the rat brain. Toxicology.

[32] Kumar M.R.R.G. (2018). Influence of age on arsenic-induced behavioral and cholinergic perturbations: Amelioration with zinc and α-tocopherol. Hum. Exp. Toxicol..

[33] Kumar N.K.K., Singh N.P. (2017). Oxidative and cellular stress as bioindicators for metal contamination in freshwater mollusk Lamellidens marginalis. Environ. Sci. Pollut. Res. Int..

[34] Srivastava P.D.Y., Gupta R., Shukla R.K., Yadav R.S., Dwivedi H.N., Pant A.B., Khanna V.K. (2018). Protective Effect of Curcumin by Modulating BDNF/DARPP32/CREB in Arsenic-Induced Alterations in Dopaminergic Signaling in Rat Corpus Striatum. Mol. Neurobiol..

[35] Kordas K.A.G., Coffman D.L., Queirolo E.I., Ciccariello D., Mañay N., Ettinger A.S. (2015). Patterns of exposure to multiple metals and associations with neurodevelopment of preschool children from Montevideo, Uruguay. J. Environ. Public Health..

[36] Rosado J.L.R.D., Kordas K., Rojas O., Alatorre J., Lopez P., Garcia-Vargas G., Del Carmen Caamaño M., Cebrián M.E., Stoltzfus R.J. (2007). Arsenic exposure and cognitive performance in Mexican schoolchildren. Environ. Health Perspect..

[37] Wasserman X.L., Faruque P., Habibul A., Pam F.-L., Alexander V.G., Vesna S., Nancy J.L., Cheng Z., Iftikhar H., Hassina M. (2004). Water Arsenic Exposure and Children’s Intellectual Function in Araihazar, Bangladesh. Environ. Health Perspect..

[38] Xi S.S.W., Wang F., Jin Y., Sun G. (2009). Transplacental and early life exposure to inorganic arsenic affected development and behavior in offspring rats. Arch. Toxicol..

[39] Jing G.Z., Liu M., Shen X., Zhao F., Wang J., Zhang J., Huang G., Dai P., Chen Y., Chen J. (2012). Changes in the synaptic structure of hippocampal neurons and impairment of spatial memory in a rat model caused by chronic arsenite exposure. NeuroToxicology.

[40] Jiang S.J., Yao S., Zhang Y., Cao F., Wang F., Li Y., Xi S. (2014). Fluoride and Arsenic Exposure Impairs Learning and Memory and Decreases mGluR5 Expression in the Hippocampus and Cortex in Rats. PLoS ONE.

[41] Luo J.-H., Qiu Z.-Q., Shu W.-Q., Zhang Y.-Y., Zhang L., Chen J.-A. (2009). Effects of arsenic exposure from drinking water on spatial memory, ultra-structures and NMDAR gene expression of hippocampus in rats. Toxicol. Lett..

[42] Luo J.-H., Qiu Z.-Q., Shu W.-Q. (2012). Arsenite exposure altered the expression of NMDA receptor and postsynaptic signaling proteins in rat hippocampus. Toxicol. Lett..

[43] Huo T.G.L.W., Zhang Y.H., Yuan J., Gao L.Y., Yuan Y., Yang H.L., Jiang H., Sun G.F. (2015). Excitotoxicity Induced by Realgar in the Rat Hippocampus: The Involvement of Learning Memory Injury, Dysfunction of Glutamate Metabolism and NMDA Receptors. Mol. Neurobiol..

[44] Sun B.-F., Yu Z.-J., Yan Y., Xiao C.-L., Kang C.-S., Guo G., Yan L., Zhu J.-D., Li Y.-M., Li Q.-M. (2015). Exercise Prevents Memory Impairment Induced by Arsenic Exposure in Mice: Implication of Hippocampal BDNF and CREB. PLoS ONE.

[45] Ramos-Chávez L.A.R.-L.C., Zepeda A., Silva-Adaya D., Del Razo L.M., Gonsebatt M.E. (2015). Neurological effects of inorganic arsenic exposure: Altered cysteine/glutamate transport, NMDA expression and spatial memory impairment. Front. Cell. Neurosci..

[46] Aung K.T.S., Maekawa F., Nohara K., Nakamura K., Tanoue A. (2015). Role of Environmental Chemical Insult in Neuronal Cell Death and Cytoskeleton Damage. Biol. Pharm. Bull..

[47] Wang C.B., Huai G., Ruolin C., Xiaoxu W., Bingwen W., Hetian J., Fengyuan P. (2015). Subchronic exposure to arsenic induces apoptosis in the hippocampus of the mouse brains through the Bcl-2/Bax pathway. J. Occup. Health.

[48] Caldwell K.E.L.M., Solomon B.R., Ali A., Allan A.M. (2015). Prenatal arsenic exposure alters the programming of the glucocorticoid signaling system during embryonic development. Neurotoxicol. Teratol..

[49] Ronchetti S.N.G., Bianchi M., Crocco M., Duvilanski B., Cabilla J. (2016). In vivoxenoestrogenic actions of cadmium and arsenic in anterior pituitary and uterus. Reproduction.

[50] Wu H.E.A.-G.N., Gharbaoui Y., Teixeira A.L., Pigott T.A. (2017). An Unusual Case of Acute Psychosis with Obsessive-Compulsive Features Following Arsenic Poisoning. J. Psychiatr. Pract..

[51] Christina R., Tyler B.R.S., Adam L.U., Andrea M.A. (2014). Fluoxetine treatment ameliorates depression induced by perinatal arsenic exposure via a neurogenic mechanism. Neurotoxicology.

[52] Avram S., Borcan F., Borcan L.C., Milac A.L., Mihailescu D. (2015). QSAR Approaches Applied to Antidepressants Induced Neurogenesis—In vivo and in silico Applications. Mini Rev. Med. Chem..

[53] Andrade C.H., Pasqualoto K.F., Ferreira E.I., Hopfinger A.J. (2010). 4D-QSAR: Perspectives in drug design. Molecules.

[54] Avram S., Maria M., Bagci E., Hritcu L., Borcan L.C., Mihailescu D. (2017). Advanced structure-activity relationships applied to *Mentha spicata* L. subsp. spicata essential oil compounds as AChE and NMDA ligands, in comparison with donepezil, galantamine and memantine—New approach in brain disorders pharmacology. CNS Neurol. Disord. Drug Targets.

[55] Damale M.G., Harke S.N., Kalam Khan F.A., Shinde D.B., Sangshetti J.N. (2014). Recent advances in multidimensional QSAR (4D–6D): A critical review. Mini Rev. Med. Chem..

[56] Cruz-Monteagudo M.S.S., Tejera E., Pérez-Castillo Y., Medina-Franco J.L., Sánchez-Rodríguez A., Borges F. (2017). Systemic QSAR and phenotypic virtual screening: Chasing butterflies in drug discovery. Drug Discov. Today.

[57] Gramatica P.E., Sangion A. (2018). QSAR modeling of cumulative environmental end-points for the prioritization of hazardous chemicals. Environ. Sci. Process. Impacts.

[58] Simões R.S.M.V., Oliveira P.R., Honorio K.M. (2018). Transfer and Multi-task Learning in QSAR Modeling: Advances and Challenges. Front. Pharmacol..

[59] Avram S., Buiu C., Duda-Seiman D., Duda-Seiman C., Borcan F., Mihailescu D. (2012). Evaluation of the Pharmacological Descriptors Related to the Induction of Antidepressant Activity and its Prediction by QSAR/QRAR Methods. Mini Rev. Med. Chem..

[60] Avram S., Buiu C., Duda-Seiman D.M., Duda-Seiman C., Mihailescu D. (2010). 3D-QSAR Design of New Escitalopram Derivatives for the Treatment of Major Depressive Disorders. Sci. Pharm..

[61] Avram S., Duda-Seiman D.M., Duda-Seiman C., Borcan F., Mihailescu D. (2010). Predicted binding rate of new cephalosporin antibiotics by a 3D-QSAR method: A new approach. Monatshefte für Chemie Chem. Mon..

[62] De Benedetti P.G., Fanelli F. (2014). Multiscale quantum chemical approaches to QSAR modeling and drug design. Drug Discovery Today.

[63] (2012). MOE (The Molecular Operating Environment).

[64] National Library of Medicine. https://sis.nlm.nih.gov/pdf/toxnetbrochure.pdf.

[65] Maciejewski A.A.C., Guo A., Marcu C., Li D., Johnson E.J., Lo J.R., Grant N., Assempour Y.D., Feunang Z., Sayeeda D.S. (2017). DrugBank 5.0: A major update to the DrugBank database for 2018. Nucleic Acids Res..

[66] Cheng F.W., Li Y., Zhou S., Jie Z., Wu G., Liu L., Tang Y. (2012). admetSAR: A Comprehensive Source and Free Tool for Assessment of Chemical ADMET Properties. J. Chem. Inf. Model..

[67] Molecular Discovery VolSurf. http://www.moldiscovery.com/software/vsplus/.

[68] Law V.C., Knox Y., Djoumbou T., Jewison A.C., Guo Y., Liu A., Maciejewski D., Arndt M., Wilson V., Neveu A. (2014). DrugBank 4.0: Shedding new light on drug metabolism. Nucleic Acids Res..

[69] Pires D.E.V., Blundell T.L., Ascher D.B. (2015). pkCSM: Predicting Small-Molecule Pharmacokinetic and Toxicity Properties Using Graph-Based Signatures. J. Med. Chem..

[70] Berman H.M.W.J., Feng Z., Gilliland G., Bhat T.N., Weissig H., Shindyalov I.N., Bourne E. (2000). The Protein Data Bank. Nucleic Acids Res..

[71] Parker L.J., Parker M.W., Morton C.J., Bocedi A., Ascher D.B., Aitken J.B., Harris H.H., Lo Bello M., Ricci G. Visualisation of Organoarsenic Human Glutathione Transferase P1-1 Complexes: Metabolism of Arsenic-based Therapeutics. https://www.rcsb.org/structure/5dak.

[72] Canaval L.R., Lutz O.M., Weiss A.K., Huck C.W., Hofer T.S. (2014). A Dissociative Quantum Mechanical/Molecular Mechanical Molecular Dynamics Simulation and Infrared Experiments Reveal Characteristics of the Strongly Hydrolytic Arsenic(III). Inorg. Chem..

[73] Roy D.R.G.S., Chattaraj P.K. (2009). Arsenic toxicity: An atom counting and electrophilicity-based protocol. Mol. Divers..

[74] Zhang Z.L.H., Zhou H., Zhu X., Zhao Z., Chi X., Shan H., Gao J. (2016). A facile route to core-shell nanoparticulate formation of arsenic trioxide for effective solid tumor treatment. Nanoscale.

[75] Teoh W.K.S.F., Shahir S. (2017). Characterization of Thiomonas delicata arsenite oxidase expressed in *Escherichia coli*. 3 Biotech.

[76] Tsai H.H.L.J., Huang J.M., Juwita R. (2013). A molecular dynamics study of the structural and dynamical properties of putative arsenic substituted lipid bilayers. Int. J. Mol. Sci..

[77] Jo S., Kim T., Iyer V.G., Im W. (2008). CHARMM-GUI: A web-based graphical user interface for CHARMM. J. Comput. Chem..

[78] Jorgensen W.L., Chandrasekhar J., Madura J.D., Impey R.W., Klein M.L. (1983). Comparison of simple potential functions for simulating liquid water. J. Chem. Phys..

[79] Kale L., Skeel R., Bhandarkar M., Brunner R., Gursoy A., Krawetz N., Phillips J., Shinozaki A., Varadarajan K., Schulten K. (1999). NAMD2: Greater scalability for parallel molecular dynamics. J. Comp. Phys..

[80] Altaf Hussain Pandith S.G., Chattaraj K. (2010). A Comparative Study of Two Quantum Chemical Descriptors in Predicting Toxicity of Aliphatic Compounds towards Tetrahymena pyriformis. Organ. Chem. Int..

[81] Dong H.M.M., Nefzi A., Houghten R.A., Giulianotti M.A., Rosen B.P. (2015). Identification of Small Molecule Inhibitors of Human As(III) S-Adenosylmethionine Methyltransferase (AS3MT). Chem. Res. Toxicol..

[82] Chen S.C.S.G., Rosen B.P., Zhang S.Y., Deng Y., Zhu B.K., Rensing C., Zhu Y.G. (2017). Recurrent horizontal transfer of arsenite methyltransferase genes facilitated adaptation of life to arsenic. Sci. Rep..

[83] Bürli R.W.H., Wei G., Ernst A., Mariga I.M., Hardern K., Herlihy A.J., Cross S.S., Wesolowski H., Chen R.D.G., McKay D.R. (2018). Novel inhibitors of As(III) S-adenosylmethionine methyltransferase (AS3MT) identified by virtual screening. Bioorg. Med. Chem. Lett..

[84] Dheeman D.S., Packianathan C., Pillai J.K., Rosen B.P. (2014). Pathway of human AS3MT arsenic methylation. Chem. Res. Toxicol..

[85] Ellison P.A.B.T., Chen F., Hong H., Zhang Y., Theuer C.P., Cai W., Nickles R.J., DeJesus O.T. (2016). High Yield Production and Radiochemical Isolation of Isotopically Pure Arsenic-72 and Novel Radioarsenic Labeling Strategies for the Development of Theranostic Radiopharmaceuticals. Bioconjug. Chem..

[86] Benjamin M.M.S.R.S., Bailey R.P., Bennett T. (1996). Sorption and filtration of metals using iron-oxide-coated sand. Water Resour..

[87] Dambies L.V.T., Guibal E. (2002). Treatment of arsenic-containing solutions using chitosan derivatives: Uptake mechanism and sorption performance. Water Resour..

[88] Perrich J.R. (1981). Activated Carbon Adsorption for Wastewater Treatment.

[89] Radovic L.R. (2000). Chemistry and Physics of Carbon.

[90] Mohan D., Singh K.P., Lehr J., Keeley J., Lehr J. (2005). Granular Activated Carbon. Water Encyclopedia: Domestc, Municipal, and Industrial Water Supply andWaste Disposal.

[91] Zhimang G.F.J., Deng B. (2005). Preparation and evaluation of GAC-based iron containing adsorbent for arsenic removal. Environ. Sci. Technol..

[92] Cortina J.L., Warshawsky A., Marinsky J.A., Marcus Y. (1997). Solvent Extraction Ion Exchange.

[93] Saha B.G.R.J., Bailey D.G., Kabay N., Arda M. (2004). Sorption of Cr(VI) from aqueous solution by Amberlite XAD-7 resin impregnated with Aliquat 336. React. Funct. Polym..

[94] Mendoza R.N.M.I.S., Vera A., Rodriguez M.A. (2000). Study of the sorption of Cr(III) with XAD-2 resin impregnated with di-(2,4,4-trimethylpentyl)phosphinic acid (Cyanex 272). Solvent Extr. Ion Exch..

[95] Muraviev D.G.L., Valiente M. (1998). Stabilization of solvent impregnated resin capacities by different techniques. React. Funct. Polym..

[96] Cotna J., Miralles N., Aguilar M., Sastre A.M. (1994). Solvent impregnated resins containing di(2-ethylhexyl) phosphoric acid. I. Preparation and stdy of the extractant on the resin. Solv. Extr. Exch..

[97] Benamor M.B.Z., Belaid T., Draa M.T. (2008). Kinetic studies on cadmium ions by Amberlite XAD7 impregnated resin containing di(20ethylhexyl) phosphoric acid as extractant. Sep. Purif. Technol..

[98] Juang R.-S. (1999). Preparation, Properties and Sorption Behavior of Impregnated Resins Containing Acidic Organophosphorus Extractants. Proc. Natl. Sci. Counc. ROC(A.).

